# Quantitative magnetic resonance imaging radiomics predicts photoreceptorness status in retinoblastoma

**DOI:** 10.1007/s00247-026-06728-6

**Published:** 2026-08-05

**Authors:** Christiaan de Bloeme, Robin Jansen, Ogul Uner, Khashayar Roohollahi, Liesbeth Cardoen, Sophia Göricke, Mériam Koob, G. Baker Hubbard, Hans Grossniklaus, Joeka de Haan, Maaike Moor, Selma Sirin, Herve Brisse, Paolo Galluzzi, Matthijs Cysouw, Josephine Dorsman, Annette Moll, Marcus de Jong, Pim de Graaf

**Affiliations:** 1 European Retinoblastoma Imaging Collaboration, Amsterdam, Netherlands; 2 Imaging and Biomarkers, Cancer Center Amsterdam, Amsterdam, Netherlands; 3 Present Address: Department of Radiology and Nuclear Medicine, Amsterdam UMC, Location VUmc, De Boelelaan 1117, Amsterdam, Noord Holland 1081HV Netherlands; 4 Department of Ophthalmology, Casey Eye Institute, Oregon Health & Science University, Portland, United States; 5 Ocular Oncology Service, Emory Eye Center, Atlanta, United States; 6 Department of Human Genetics, Amsterdam UMC, Location VUmc, Amsterdam, Netherlands; 7 Department of Radiology, Institute Curie, Paris, France; 8 Department of Diagnostic and Interventional Radiology and Neuroradiology, University Hospital Essen, Essen, Germany; 9 Department of Radiology, Centre Hospitalier Universitaire Vaudois (CHUV) and University of Lausanne, Lausanne, Switzerland; 10 Department of Diagnostic Imaging, University Children’s Hospital Zurich, Zurich, Switzerland; 11 Unit of NeuroImaging and NeuroIntervention, Azienda Ospedaliera Universitaria Santa Maria alle Scotte, Siena, Italy; 12 Department of Ophthalmology, Amsterdam UMC, Location VUmc, Amsterdam, Netherlands

**Keywords:** Head and neck, Magnetic resonance imaging, Photoreceptorness, Radiogenomics, Radiomics, Retinoblastoma

## Abstract

**Background:**

Molecular characteristics of retinoblastoma cannot be assessed before treatment because tumor biopsy is contraindicated. Photoreceptorness reflects photoreceptor-related gene expression and tumor differentiation. Non-invasive imaging biomarkers that capture this biology are therefore needed.

**Objective:**

To evaluate whether quantitative radiomics derived from pretreatment magnetic resonance imaging can predict loss of photoreceptorness in retinoblastoma and validate this approach in an independent cohort.

**Materials and methods:**

In this retrospective multicenter study, patients with retinoblastoma who underwent primary enucleation and had both pretreatment T2-weighted magnetic resonance imaging and genome-wide messenger RNA expression data were included. Tumors in the highest and lowest photoreceptorness quartiles were analyzed. Whole-tumor segmentations were used to extract radiomic features with PyRadiomics. Multiple machine-learning pipelines were evaluated using repeated stratified cross-validation, and the best-performing model was tested in an independent cohort.

**Results:**

Forty-five patients (median age, 18 months [range, 2–70], 18 female) were included: 29 in the training cohort and 16 in the independent testing cohort. The best-performing model used recursive feature elimination with a random forest classifier and achieved a mean cross-validated area under the receiver operating characteristic curve of 0.83 in the training cohort. In the independent testing cohort, the model achieved an area under the receiver operating characteristic curve of 0.81 (95% confidence interval, 0.54–1.00) for predicting loss of photoreceptorness.

**Conclusion:**

Quantitative magnetic resonance imaging radiomics showed preliminary moderate-to-good discriminatory performance for non-invasively predicting photoreceptorness status in retinoblastoma. These proof-of-concept findings suggest that radiomics may capture imaging features related to molecular tumor differentiation and could support the development of personalized treatment strategies.

**Graphical abstract:**

**Figure:**
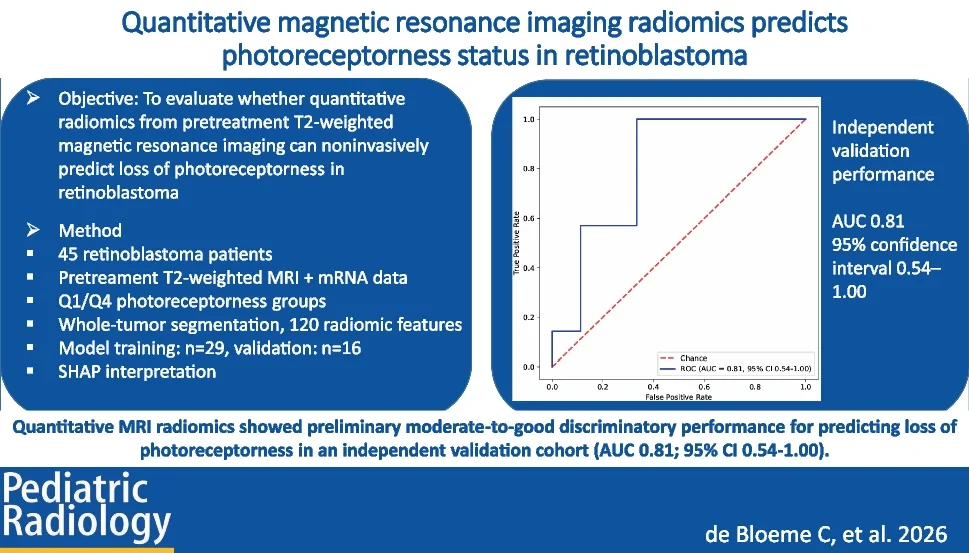

**Supplementary Information:**

The online version contains supplementary material available at https://doi.org/10.1007/s00247-026-06728-6.

## Introduction

Retinoblastoma is a rare disease, with an incidence of 1 case per 17,000 live births, yet it remains the most common primary intraocular cancer in childhood [1, 2]. While its clinical presentation has long been recognized, recent advances in molecular profiling have begun to unravel the biological heterogeneity underlying this malignancy. One such discovery is the concept of *photoreceptorness*, which describes the extent to which retinoblastoma cells express genes typically active in retinal photoreceptor cells [3, 4]. This gene signature reflects the degree of cellular differentiation within the tumor and has emerged as an indicator of disease progression. Tumors with high photoreceptorness tend to resemble normal retinal tissue and are often smaller, more localized, and diagnosed at a younger age [3, 5]. In contrast, tumors with low or loss of photoreceptorness display features of dedifferentiation, including diffuse growth, multifocality, and increased genomic instability [3]. Notably, loss of photoreceptorness has also been associated with distinctive ex vivo chemotherapy sensitivity [4], suggesting that molecular differentiation may influence therapeutic response. These findings position photoreceptorness not merely as a marker of tumor subtype, but as a dynamic trait that evolves during tumor progression and may inform prognosis and treatment strategies.

Despite these molecular insights, retinoblastoma continues to be treated without preceding histopathologic or genetic confirmation, as direct biopsy is contraindicated due to the risk of extraocular tumor dissemination. This constraint compels clinicians to rely on ophthalmologic assessment and radiological imaging, which do not capture the underlying molecular heterogeneity that could guide personalized therapy. This challenge is amplified by the current emphasis on eye-preserving treatments, such as intravitreal chemotherapy and selective intra-arterial chemotherapy (SIAC), which are increasingly utilized even in more advanced cases [6–8]. While clinically advantageous, this shift further limits the possibility of obtaining unaltered genetic data for personalizing patient treatment and for research.

To address this gap, an emerging field known as radiogenomics seeks to correlate radiological imaging features with genomic data. Radiogenomic analyses may be based on qualitative imaging traits evaluated visually by radiologists, such as multifocality or globe-filling lesions as seen in the retinoblastoma imaging atlas [9], or on quantitative features extracted via computational radiomics, such as texture or shape-based imaging features. Initial work by Jansen et al. [9] demonstrated that qualitative MRI features could be linked to whole-genome expression profiles, including the photoreceptorness gene signature. Specifically, loss of photoreceptorness was associated with advanced-stage imaging features, such as multifocality, tumors encompassing the entire retina, and globe-filling lesions. These radiogenomic associations were subsequently validated in an independent multicenter cohort, reinforcing the potential of MRI-based imaging traits to non-invasively reflect underlying molecular characteristics [5].

However, qualitative radiogenomics is inherently limited by intra- and interobserver variability and the need for expert radiologists, particularly in rare diseases like retinoblastoma. These limitations can be mitigated by applying quantitative radiomics, which uses mathematical algorithms to extract reproducible imaging features independent of visual interpretation, thereby reducing observer dependency and providing a more objective and scalable approach to non-invasive molecular profiling [10].

The objective of this study is to assess the diagnostic accuracy of quantitative radiomics applied to pretreatment MR imaging for predicting the loss of photoreceptorness, followed by validation of these predictions in an independent dataset.

## Methods

This retrospective multicenter investigation received approval from the institutional review board of Amsterdam UMC, location VUmc (IRB number IRB00002991) with a waiver of informed consent.

### Patients

This study used two patient cohorts from previously published studies [3, 9, 11, 12]. Patients from these cohorts were included when (1) pretreatment T2-weighted images were available and (2) histopathology and subsequent genome-wide mRNA expression data were available. Patients were excluded if genome-wide mRNA expression profiling identified their tumors as *MYCN*-amplified *RB1*-wildtype retinoblastomas—a distinct molecular carcinogenic entity driven by *MYCN* activation rather than by *RB1* loss—since this study focuses on *RB1*-associated tumorigenesis [13].

The first cohort of patients was a consecutive patient series from a single tertiary referral center specialized in retinoblastoma care, Amsterdam UMC, Amsterdam, the Netherlands, obtained from April 1995 to February 2012, referred to as the model training cohort [3, 9, 11].

The second cohort of patients was a retrospective multicenter cohort study collected from two tertiary referral centers specialized in retinoblastoma care: (1) Emory Eye Center, Atlanta, Georgia, USA [April 2005 to December 2011] and (2) Amsterdam UMC, Amsterdam, the Netherlands [December 2015 to October 2018], referred to as model testing cohort [5].

For each center within each cohort, patients were grouped into quartiles based on their level of photoreceptorness score, with Q1 representing the top 25% of tumors with the highest photoreceptorness score, and Q4 representing the bottom 25% with the lowest photoreceptorness score. Only patients falling within the highest (Q1) or lowest (Q4) quartiles of photoreceptorness were included in this study as phenotypic anchors, capturing the molecular extremes of differentiation to maximize contrast and enhance the discriminative power of our radiomics‐based prediction models.

### Gene expression data processing and analysis

#### Model training cohort

##### Gene expression data Amsterdam UMC

Gene expression data were acquired through microarray expression analysis, followed by normalization of absolute expression values using robust multi-chip array normalization via the Affy Bioconductor package (Bioconductor, Seattle, WA), and subsequently log2-transformed [14]. In cases where a gene was targeted by multiple probes, only the probe closest to the 3′-end was used for analysis, as per previous methodology [3]. Additionally, radiomic features were evaluated for correlation with a predefined gene expression profile related to photoreceptorness [3].

#### Model testing cohort

To minimize non-biological variations due to platform and procedural differences, expression data from each referral center were separately processed, analyzed, and merged using scaling adjustments like *Z*-score for cross-center downstream analysis.

##### Gene expression data Amsterdam UMC

Gene expression data were derived from whole-genome total RNA sequencing of the retinoblastoma samples. Total RNA extraction was conducted using a dual RNA/DNA isolation protocol from tissue preserved in OCT (AllPrep DNA/RNA—QIAGEN, Hilden, Germany). Library preparation was accomplished using the SMARTer Stranded Total RNA-Seq kit v3 (Takara Bio USA, San Jose, CA). Paired-end sequencing of RNA samples was carried out on the NovaSeq 6000 system (Illumina, San Diego, CA) at 60× coverage with 100-bp fragment sizes. Subsequently, the FASTQ files underwent cleaning using fastp [15]. Sequence alignment to the human reference genome 38 (hg38) was performed using HISAT2 [16], followed by BAM conversion, sorting, and indexing with SAMTOOLS [17]. Gene-level reads were counted using featureCounts [18], with multi-mapping reads incorporated via fractional counting. Expression analysis was conducted using edgeR [19], with original library size normalized to effective library size by the trimmed mean of *M*-value (TMM).

##### Gene expression data Emory Eye Center

Transcriptomic data were generated through microarray experiments using GeneChip Human Gene 2.0 ST and Human Genome U133A arrays (Affymetrix/Thermo Fisher Scientific, Santa Clara, CA). CEL files from Emory Eye Center were imported into expression objects using the oligo and Affy packages, respectively [14, 20]. Subsequently, data normalization was performed using robust multi-chip average (RMA) via the Limma package [21].

### Quantitative magnetic resonance imaging feature extraction

To enable quantitative feature extraction from MR images for both the first and second cohort, one independent reviewer (C.M.d.B.) manually delineated the whole tumor on T2-weighted MR images using 3D Slicer (version 4.10.1, MIT, Boston, MA) [22]. The delineation excluded non-enhancing areas of pure retinal detachment and subretinal fluid or hemorrhage and was validated by expert radiologists (P.d.G and M.C.d.J.). If needed, segmentations were adjusted based on consensus. Image processing and feature calculations were conducted with PyRadiomics (an open-source Python package for radiomic feature extraction, version 3.1.0) [10]. Prior to feature extraction, MR images and delineations were resampled to 2×2×2 mm isotropic voxels using B-spline interpolation, and grayscale intensities were discretized using a fixed bin width of 64. C extensions were enabled. Only original image features were extracted, including first-order intensity features, shape-based features, and texture features derived from GLCM, GLRLM, GLSZM, NGTDM, and GLDM matrices [10]. Subsequently, 120 radiomics features were extracted from the delineated tumors, categorized into three main categories: intensity (*n*=19), morphology (*n*=26), and texture (*n*=75). These radiomic features were evaluated for correlation with a predefined gene expression profile related to photoreceptorness extracted from the RNA expression data [3].

### Model development for predicting loss of photoreceptorness in quartiles 1 and 4

The model to predict loss of photoreceptorness only employed the training cohort.

To develop the most effective model for predicting the loss of photoreceptorness, a previously established framework was used, following the approach described by de Bloeme et al. [23, 24].

This framework employs a repeated cross-validation strategy to evaluate different combinations of feature selection techniques, dimensionality reduction methods, and classification algorithms to derive an average classification performance for each configuration.

Feature preprocessing involved *z*-score normalization, and highly correlated features (Pearson’s *r*>0.8) were removed to minimize redundancy, prioritizing the feature with the highest average correlation within the dataset. Three distinct dimensionality reduction techniques were applied: recursive feature elimination using logistic regression (RFE-LR) and random forests (RFE-RF), as well as a univariate feature selection method based on the *F*-statistic from ANOVA, selecting the top 10th percentile of features. Given the balanced nature of the dataset—patients were categorized into quartile-based groups—no oversampling techniques were required.

For classification, two models were implemented: logistic regression and a widely used non-parametric ensemble method, the random forest classifier [25]. Hyperparameter tuning was conducted using nested cross-validation. Model generalizability was assessed through a stratified fivefold cross-validation procedure, where 80% of the data was used for training and the remaining 20% for validation within each fold. This process was repeated 20 times to mitigate variability due to random sampling. Performance evaluation metrics included the area under the receiver operating characteristic curve (AUC), with mean AUC and standard deviation (SD) reported across all iterations. Additionally, model calibration was quantified using the Brier score, with 0.0 indicating optimal performance and 1.0 the worst performance [26].

To interpret the contributions of individual features in the best-performing model, SHapley Additive exPlanations (SHAP) values were computed from a model trained on the training cohort [27]. This final model was subsequently applied for validation.

### Model validation for predicting loss of photoreceptorness in quartiles 1 and 4

A separate cohort was used to validate the best-performing model in predicting loss of photoreceptorness. Performance of this applied best-performing model was evaluated using the receiver operating characteristic curve, and the area under the curve (AUC) was calculated. To estimate uncertainty related to the small sample size, a 95% confidence interval for the AUC in the independent testing cohort was calculated using 2,000 bootstrap resamples.

### Statistical analysis of clinical and magnetic resonance imaging acquisition variables

Clinical characteristics were compared between tumors with high (Q1) and low (Q4) photoreceptorness within each cohort and between the training and independent testing cohorts to assess potential cohort heterogeneity. Evaluated variables included sex, laterality, center, age at MRI, and MRI examination year; magnetic field strength was additionally assessed for the cohort comparison. Magnetic field strength was extracted from DICOM metadata of the T2-weighted sequences and categorized by scanner field strength. Continuous variables are reported as median [IQR] (range) and categorical variables as *n* (%). Group differences were assessed using the Mann-Whitney *U* test for continuous variables and Fisher’s exact test or Fisher-Freeman-Halton exact test for categorical variables. *P*-values were not calculated for constant variables, and two-sided *P*-values<0.05 were considered statistically significant.

## Results

### Patients

A total of 45 patients were included in this study, with 29 in the training cohort and 16 in the testing cohort. The training cohort consisted of 14 patients with high photoreceptorness (Q1) and 15 with low photoreceptorness (Q4), while the testing cohort included 9 patients with high photoreceptorness and 7 with low photoreceptorness. In both the training and testing cohorts, the median patient age was statistically significantly older in tumors showing a low photoreceptorness score. In the training cohort, the median age was 10 months for the high and 33 months for the low photoreceptorness patients (*P*<0.001). In the testing cohort, the median age was 5 months for the high and 27 months for the low photoreceptorness patients (*P*<0.01). Clinical parameters for patients in each cohort are summarized in Table 1. A summary of imaging parameters used for this study is shown in Supplementary Material 1 (Supplementary Material 1: Overview of the imaging parameters of T2-weighted sequences used in this study).

**Table 1:** Clinical parameters for the included patients for each cohort

Training cohort	Total patients (*n*=29)	High photoreceptorness [Q1] (*n*=14)	Low photoreceptorness [Q4] (*n*=15)	*P*-value
Female sex, *n* (%)	9 (31%)	3 (21.4%)	6 (40%)	0.43^a^
Laterality (bilateral), *n* (%)^d^	8 (27.6%)	6 (42.9%)	2 (13.3%)	0.11^a^
Center (Amsterdam UMC),* n* (%)	29 (100%)	14 (100%)	15 (100%)	NC^c^
Age in months at scan date; median [IQR] (range)	18 [10–37.50] (2–70)	10 [4.75–13.25] (2–20)	33.0 [24.0–33.0] (10–70)	<0.001^b^
MRI examination year; median [IQR] (range)	2003 [2000–﻿2008] (1996-2012)	2000.5 [1998.75–2000.50] (1996-2010)	2007 [2002–﻿2010] (1996–﻿2012)	0.03^b^
Testing cohort	Total patients (*n*=16)	High photoreceptorness [Q1] (*n*=9)	Low photoreceptorness [Q4] (*n*=7)	*P*-value
Female sex, *n* (%)	9 (56.3%)	6 (66.7%)	3 (42.9%)	0.62^a^
Laterality (bilateral), *n* (%)^d^	2 (12.5%)	2 (22.2%)	0 (0.0%)	0.48^a^
Center (Amsterdam UMC),* n *(%)	7 (43.8%)	4 (44.4%)	3 (42.9%)	1.000^a^
Age in months at scan date; median [IQR] (range)	14 [4–25.5] (2–46)	5 [2–14.0] (2–17)	27.0 [19.0–41.0] (4–46)	<0.01^b^
MRI examination year; median [IQR] (range)	2010.5 [2006–﻿2017] (2005-2018)	2010 [2005.5–2017] (2005–﻿2018)	2011 [2006–﻿2011] (2005–﻿2017)	0.87^b^

Data are presented as *n* (%) or median [*IQR* interquartile range] (range). *P*-values were derived from ^a^Fisher’s exact test or ^b^Mann-Whitney *U* test

^c^Not computable as center is a constant

^d^Of three bilateral retinoblastoma patients, both eyes were enucleated and included in the analysis

*MRI* magnetic resonance imaging

Clinical characteristics and MRI acquisition parameters were compared between the training and independent testing cohorts (Table 2). Sex, laterality, and age at MRI did not differ significantly between cohorts. MRI examination year differed significantly, with more recent examinations in the testing cohort than in the training cohort (median, 2010.5 vs 2003; *P*<0.001). Magnetic field strength also differed significantly (*P*<0.001), with 3.0-T scans present only in the testing cohort (8/16, 50.0%).

**Table 2:** Comparison of clinical characteristics and magnetic resonance imaging acquisition parameters between the training and independent testing cohorts

	Training cohort (*n*=29)	Testing cohort (*n*=16)	*P*-value
Female sex, *n* (%)	9 (31.0%)	9 (56.3%)	0.12^a^
Laterality (bilateral), *n* (%)	8 (27.6%)	2 (12.5%)	0.29^a^
Age in months at scan date; median [IQR], range	18 [10–37.5] (2–70)	14 [4–25.5] (2–46)	0.18^b^
MRI examination year; median [IQR], range	2003 [2000–﻿2008] (1996–﻿2012)	2010.5 [2006–﻿2017] (2005–﻿2018)	<0.001^b^
Magnetic field strength, *n* (%)			<0.001^c^
0.95 T	4 (13.8%)	0 (0.0%)	
1.5 T	25 (86.2%)	8 (50.0%)	
3.0 T	0 (0.0%)	8 (50.0%)	

Data are presented as *n* (%) or median [*IQR* interquartile range] (range). *P*-values were derived from ^a^Fisher’s exact test, ^b^Mann-Whitney *U* test, or ^c^Fisher-Freeman-Halton exact test

*MRI* magnetic resonance imaging

### Best-performing model based on the training cohort for predicting loss of photoreceptorness in quartiles 1 and 4

**Fig. 1:**
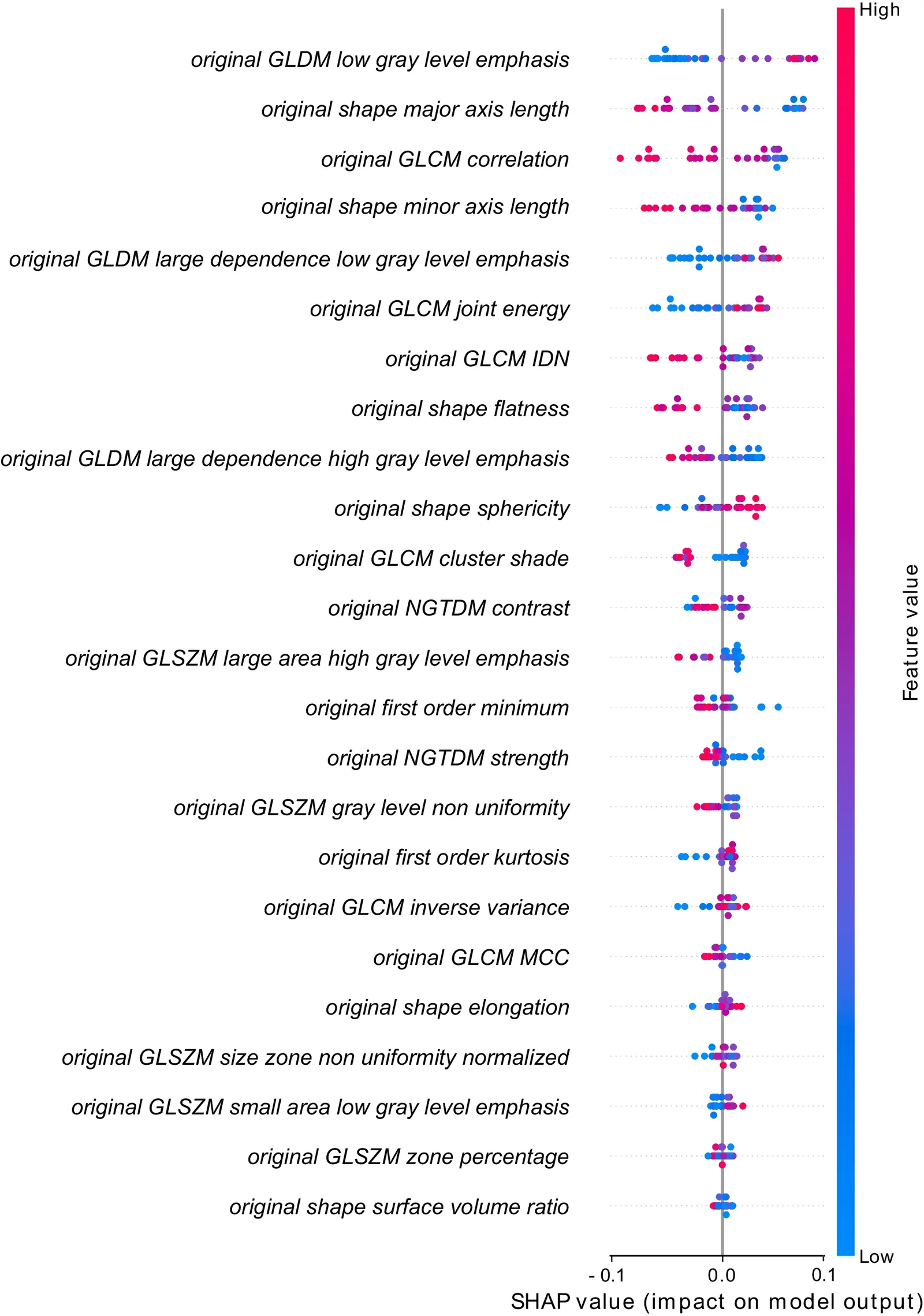
SHapley Additive exPlanations values for the best-performing model using recursive feature elimination with random forest and a random forest classifier for predicting loss of photoreceptorness in quartiles 1 and 4 of the training cohort. Loss of photoreceptorness was defined as the positive class. *Positive SHAP values* indicate a shift in model prediction toward low photoreceptorness, whereas *negative SHAP values* indicate a shift toward high photoreceptorness. Among the top contributing features, higher values of *original GLDM low gray level emphasis* and *original GLDM large dependence low gray level emphasis* shifted predictions toward low photoreceptorness. Conversely, lower values *of original GLCM correlation* and lower values of the shape features *original shape major axis length* and *original shape minor axis length* shifted predictions toward low photoreceptorness

The best-performing combination of methods used recursive feature elimination with random forest (RFE-RF) and a random forest classifier, resulting in a mean AUC of 0.83 (95% CI, 0.78–1.00; SD 0.15) with a mean Brier score of 0.18 (SD 0.06). The other combinations of methods had AUCs ranging from 0.75 (SD 0.25) to 0.83 (SD 0.18). Performances of all created radiomics models are shown in Table 3. For the best-performing combinations of methods, Fig. 1 shows the SHAP values of the most influential features, with texture features playing the dominant role. Loss of photoreceptorness was defined as the positive class in the SHAP analysis. Therefore, positive SHAP values indicate a shift in model prediction toward low photoreceptorness. Higher values of *original GLDM low gray level emphasis* and *original GLDM large dependence low gray level emphasis*, and lower values of *original GLCM correlation*, shifted predictions toward low photoreceptorness. All features can be found in Supplementary Material 2 (Supplementary Material 2: Summary of all features for the best-performing model for predicting loss of photoreceptorness in quartiles 1 and 4 in the training cohort).

**Table 3:** Performance of radiomics models predicting loss of photoreceptorness in quartiles 1 and 4 of the training cohort

Feature selection	Classifier	Mean AUC	AUC 95% CI	AUC SD	Mean Brier score	Brier score SD
RFE-RF	Random forest	0.83	0.78–1.00	0.15	0.18	0.06
None	Random forest	0.83	0.75–1.00	0.16	0.18	0.06
RFE-LR	Random forest	0.81	0.67–1.00	0.16	0.18	0.06
Univariate	Logistic regression	0.81	0.67–1.00	0.19	0.21	0.11
RFE-LR	Logistic regression	0.76	0.67–0.89	0.18	0.24	0.12
Univariate	Random forest	0.76	0.67–0.89	0.19	0.22	0.1
RFE-RF	Logistic regression	0.76	0.67–0.89	0.19	0.25	0.14
None	Logistic regression	0.75	0.67–0.89	0.19	0.25	0.13

Results from all cross-validation analyses

*Univariate*, a univariate selection method based on ANOVA testing that retained the top 10 percentile features; *AUC*, area under the receiver operating characteristic curve; *CI* confidence interval, *RFE-LR* recursive feature elimination using logistic regression, *RFE-RF* recursive feature elimination using random forest, *SD* standard deviation; Brier score assesses the model calibration and refinement (0.0 being optimal)

Features in three categories were assessed: intensity features, texture features, and morphology features. Intensity features comprised first-order statistics [19 features]. Morphology features comprised shape-based two-dimensional features [10 features] and three-dimensional features [16 features]. Texture features comprised features based on gray-level co-occurrence matrix [24 features], gray-level dependence matrix [14 features], gray-level run length matrix [16 features], gray-level size zone matrix [16 features], and neighborhood gray-tone difference matrix [5 features]. *SHAP* SHapley Additive exPlanations; *GLCM* gray-level co-occurrence matrix; *GLDM* gray-level dependence matrix; *GLRLM* gray-level run length matrix; *GLSZM* gray-level size zone matrix; *NGTDM* neighborhood gray-tone difference matrix.

### Validation of the model on the independent testing cohort for predicting loss of photoreceptorness

**Fig. 2:**
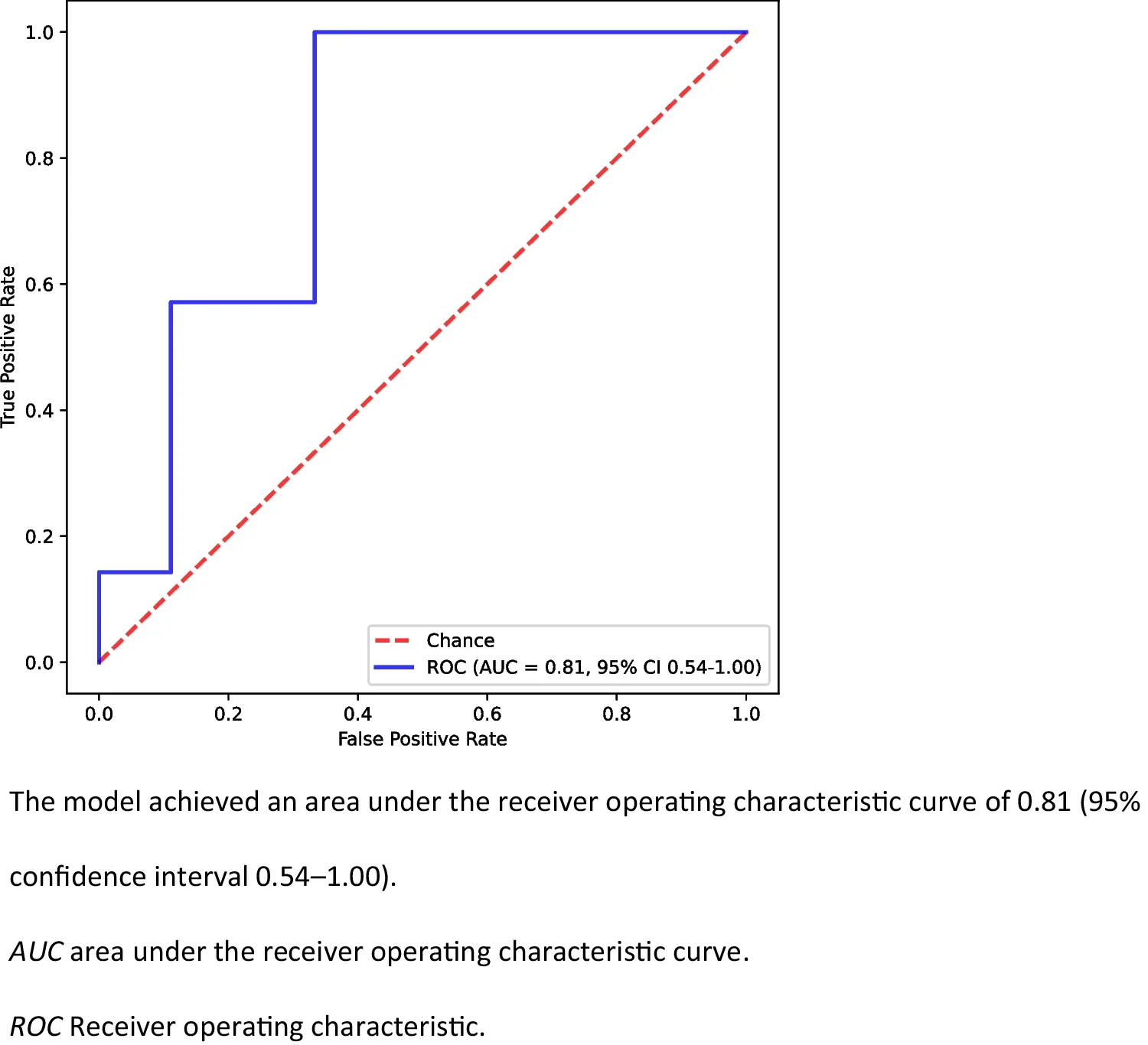
Receiver operating characteristic curve of the best-performing model using recursive feature elimination with random forest and a random forest classifier in the independent testing cohort

Applying the best-performing model using recursive feature elimination with random forest (RFE-RF) and a random forest classifier on our independent testing cohort resulted in an AUC of 0.81 (95% CI, 0.54–1.00), indicating preliminary moderate-to-good discriminatory performance, although with substantial uncertainty as reflected by the wide confidence interval (Fig. 2).

## Discussion

In this multicenter proof-of-concept study, quantitative MRI radiomics showed preliminary moderate-to-good discriminatory performance for predicting photoreceptorness status in retinoblastoma. The best-performing model, based on recursive feature elimination with random forest classification, achieved a mean cross-validated AUC of 0.83 in the training cohort and an AUC of 0.81 in the independent testing cohort, although the wide 95% confidence interval of 0.54–1.00 indicates substantial statistical uncertainty. The observed differences in MRI examination year and magnetic field strength indicate temporal and scanner-related heterogeneity between cohorts. Although independent testing performance remained comparable to the cross-validated training performance, this heterogeneity may still have influenced radiomic feature distributions and should be considered when interpreting model generalizability. These results suggest that radiomics features extracted from pretreatment T2-weighted MRI may encode biologically relevant information about tumor differentiation. SHAP analyses revealed that texture features were the most influential predictors. Several texture-based features associated with image heterogeneity contributed more strongly to predictions of low photoreceptorness, suggesting increased intratumoral heterogeneity in less differentiated tumors. Although the biological interpretation of individual radiomics texture features remains indirect and requires further validation, these findings are consistent with the observation that tumors with low photoreceptorness occurred in significantly older patients across both cohorts, supporting an association between increasing age at diagnosis and transcriptional dedifferentiation in retinoblastoma.

Our findings extend the qualitative radiogenomic work of Jansen et al. [9], who first reported that low photoreceptorness was associated with advanced MRI features such as multifocality, diffuse intraretinal tumor growth, and globe-filling tumors. These associations were subsequently validated in an independent multicenter cohort [5]. Additionally, radiomics has been successfully applied to identify other molecular subtypes and to improve the detection of postlaminar optic nerve invasion (PLONI) [10, 23, 24, 28]. As demonstrated in a recent publication [24], radiomics was able to differentiate *MYCN*-amplified *RB1*-wildtype retinoblastomas from biallelic *RB1*-mutant tumors, achieving moderate performance with an AUC of 0.64, highlighting the potential of imaging to capture distinct genetic drivers. Similarly, radiomics enhanced prediction of PLONI, achieving an AUC of 0.90 [28]. Together, these studies underscore that radiomics is not only capable of reflecting differentiation states such as photoreceptorness but may also serve as a broader tool for genotype–phenotype mapping in retinoblastoma.

The ability to non-invasively infer photoreceptorness status has several potential clinical applications. First, photoreceptorness reflects tumor differentiation and progression [3], and its loss has been linked to distinct chemotherapy sensitivity profiles [4]. Thus, radiomics-based prediction could support risk stratification and treatment tailoring in an era where eye-sparing therapies such as selective intra-arterial chemotherapy and intravitreal chemotherapy are increasingly used [6–8]. Second, radiomics may complement emerging liquid biopsy approaches from aqueous humor [29], together providing a multimodal framework for pretreatment molecular profiling [30–32]. Notably, low photoreceptorness correlates with dedifferentiation, an increase in somatic copy number alterations, and an increase in molecular changes [3, 33]. This raises the possibility that tumors in the lowest quartile (Q4) may harbor more genomic alterations as potential drivers of tumor progression, such as gains and amplifications on 1q (*MDM4*-gene), 2p (*MYCN*), 6p [34, 35], or mutations in BCL-6 Corepressor (*BCOR*) gene [33, 36, 37]. Radiomics could potentially aid in identifying such high-risk tumors within this subgroup, though this requires further investigation.

### Limitations

This study has several limitations. Firstly, the sample size was relatively small, reflecting the rarity of retinoblastoma and the challenges of obtaining both MRI and genome-wide expression data. Although this is one of the largest radiogenomic analyses in this disease to date, the modest number of patients limits the statistical stability of the observed AUC. In particular, the independent validation AUC of 0.81 had a wide 95% confidence interval of 0.54–1.00, reflecting substantial statistical uncertainty. This may restrict the generalizability of our findings. Therefore, the reported performance should be interpreted as preliminary and hypothesis-generating rather than definitive evidence of robust generalizability, and requires validation in larger, preferably prospective multicenter cohorts. Secondly, the retrospective design of both cohorts introduces the possibility of selection bias, as only patients with available imaging and molecular data could be included. In addition, the study population consisted exclusively of patients who underwent enucleation, reflecting more advanced disease. Thirdly, imaging protocols varied across centers and scanners, which may have influenced radiomics feature extraction despite our efforts to harmonize data through resampling and normalization. This was supported by the cohort comparison, which showed significant differences in MRI examination year and magnetic field strength between the training cohort and independent testing cohort. Such heterogeneity is inherent to multicenter studies but underscores the need for prospective harmonized imaging protocols in future work [38]. No formal feature stability analysis or feature-level scanner harmonization method, such as ComBat, was performed; therefore, the robustness of the selected radiomic features across scanner field strength, slice thickness, and voxel size remains uncertain.

Another limitation relates to the dichotomization of patients into the highest and lowest quartiles of photoreceptorness. While this approach maximized contrast and improved model discrimination, it excluded patients with intermediate levels of photoreceptorness, thereby limiting the applicability of our model across the full biological spectrum of the disease. Methodological reproducibility is another consideration, as segmentation reproducibility was not quantitatively assessed, and potential segmentation-related variability may have influenced radiomic feature extraction and model performance. Both eyes from three bilateral retinoblastoma patients were included in the analysis, which may have introduced non-independence of observations and potential bias. No sensitivity analysis excluding one eye per bilateral patient was performed. Finally, while SHAP analyses provided insight into the most influential radiomics features, the biological interpretation of these mathematical descriptors remains indirect, and further work is needed to link specific radiomics patterns to histopathological correlates.

## Conclusion

In summary, this proof-of-concept study shows that quantitative magnetic resonance imaging radiomics can non-invasively predict photoreceptorness status in retinoblastoma, with preliminary moderate-to-good discriminatory performance across an independent cohort. By capturing intratumoral heterogeneity linked to molecular differentiation, radiomics provides a reproducible and scalable alternative to qualitative radiogenomics. These findings highlight the potential of radiomics not only as a surrogate for photoreceptorness but also as a tool to differentiate underlying molecular differences in retinoblastoma, possibly paving the way toward personalized, image-guided treatment strategies in retinoblastoma.

## Supplementary Information

Below is the link to the electronic supplementary material.

ESM1 (DOCX 25.6 KB)

## Data Availability

The datasets analysed during the current study are not publicly available due to privacy and ethical restrictions.
