# Supplementary material for: Quantitative magnetic resonance imaging radiomics predicts photoreceptorness status in retinoblastoma
Source: Pediatr Radiol. 2026 Aug 5;56(12):2731–41. doi: 10.1007/s00247-026-06728-6 (PMC13759574; doi:10.1007/s00247-026-06728-6)
Supplement: Supplementary file 1 [file 247_2026_6728_MOESM1_ESM.docx]

# Supplemental Materials

Supplementary material 1: Overview of the imaging parameters of T2-weighted sequences used in this study.

| Sequence | Slice thickness | Pixel Dimensions | Echo-Time | Repetition-Time | Scanners (Tesla) |
| --- | --- | --- | --- | --- | --- |
| 2D T2 | 3.00, 2.00-6.50 | 0.49x0.49, 0.27x0.27-1.00x1.00 | 120.0, 60.0-152.3 | 2470.0, 2000.0-8002.0 | 7 GE Medical Systems Discovery MR750 (3.0T),  2 GE Medical Systems Signa Genesis (1.5T),  5 GE Medical Systems Signa Excite (1.5T),  1 Siemens Aera (1.5T),  4 Siemens Magnetom Expert (1.0T),  14 Siemens Magnetom Vision (1.5T),  13 Siemens Sonata (1.5T),  1 Siemens TrioTim (3.0T) |

Note.- Data are presented as median and range or number of scans performed on each scanner (Tesla). T2 = T2-weighted MR sequences. Discovery MR750, Signa Genesis, and Signa Excite, GE Healthcare, Chicago, IL. Aera, Magnetom Expert, Magnetom Vision, Sonata, TrioTim, Siemens, Erlangen, Germany.

Supplementary material 1: Summary of all features for the best-performing model for predicting loss of photoreceptor in quartiles 1 and 4 in the training cohort.

| **Feature** | **Method** | **Feature category** |
| --- | --- | --- |
| *original GLDM low gray level emphasis* | GLDM | Texture |
| *original shape major axis length* |  | Morphology |
| *original GLCM correlation* | GLCM | Texture |
| *original shape minor axis length* |  | Morphology |
| *original GLDM large dependence low gray level emphasis* | GLDM | Texture |
| *original GLCM joint energy* | GLCM | Texture |
| *original GLCM IDN* | GLCM | Texture |
| *original shape flatness* | Shape | Morphology |
| *original GLDM large dependence high gray level emphasis* | GLDM | Texture |
| *original shape sphericity* |  | Morphology |
| *original GLCM cluster shade* | GLCM | Texture |
| *original NGTDM contrast* | NGTDM | Texture |
| *original GLSZM large area high gray level emphasis* | GLSZM | Texture |
| *original first order minimum* |  | Intensity |
| *original NGTDM strength* | NGTDM | Texture |
| *original GLSZM gray level non uniformity* | GLSZM | Texture |
| *original first order kurtosis* |  | Intensity |
| *original GLCM inverse variance* | GLCM | Texture |
| *original GLCM MCC* | GLCM | Texture |
| *original shape elongation* |  | Morphology |
| *original GLSZM size zone non uniformity normalized* | GLSZM | Texture |
| *original GLSZM small area low gray level emphasis* | GLSZM | Texture |
| *original GLSZM zone percentage* | GLSZM | Texture |
| *original shape surface volume ratio* |  | Morphology |

Note.— *GLCM* gray-level co-occurrence matrix, *GLDM* gray-level dependence matrix, *GLSZM* gray-level size zone matrix, *NGTDM* neighborhood gray-tone difference matrix
